# Aromatic Amide Foldamers Show Conformation‐Dependent Electronic Properties

**DOI:** 10.1002/cphc.202500672

**Published:** 2025-11-08

**Authors:** Rajarshi Samajdar, Xiaolin Liu, Kazusa Kuyama, Yui Kidokoro, Fumi Takeda, Iwao Okamoto, Masatoshi Kawahata, Kosuke Katagiri, Jeffrey S. Moore, Aya Tanatani, Charles M. Schroeder

**Affiliations:** ^1^ Department of Chemical and Biomolecular Engineering University of Illinois at Urbana‐Champaign Urbana 61801 Illinois; ^2^ Beckman Institute for Advanced Science and Technology University of Illinois at Urbana‐Champaign Urbana 61801 Illinois; ^3^ Department of Chemistry University of Illinois at Urbana‐Champaign Urbana 61801 Illinois; ^4^ Department of Chemistry Faculty of Science Ochanomizu University 2‐1‐1 Otsuka, Bunkyo‐ku Tokyo 112‐8610 Japan; ^5^ Faculty of Pharmaceutical Science Showa Pharmaceutical University 3‐2‐1 Higashitamagawagakuen, Machida Tokyo 194‐8543 Japan; ^6^ Department of Chemistry of Functional Molecules Faculty of Science and Engineering Konan University 8‐9‐1 Okamoto, Higashinada, Kobe Hyogo 658‐8501 Japan; ^7^ Department of Materials Science and Engineering University of Illinois at Urbana‐Champaign Urbana 61801 Illinois; ^8^ Present address: Department of Chemical and Biological Engineering Princeton University 41 Olden Street Princeton 08544 New Jersey

**Keywords:** electron transport, electron tunneling, foldamers, molecular electronics, supramolecular chemistry

## Abstract

Electron transport in organic molecules and biomolecules is governed by electronic structure and molecular conformations. Despite recent progress, key challenges remain in understanding the role of intramolecular interactions and three‐dimensional (3D) conformations on the electron transport behavior of organic molecules. In this work, the electronic properties of aromatic amide foldamers are characterized that organize into distinct 3D structures, including an extended secondary amide that adopts a *trans*‐conformation and a folded *N*‐methylated tertiary amide that adopts a *cis*‐conformation. Results from single‐molecule electronic experiments show that the extended secondary amide exhibits a fourfold enhancement in molecular conductance compared to the folded *N*‐methylated tertiary amide, despite a longer contour length. The results show that extended amide molecules are governed by a through‐bond electron transport mechanism, whereas folded amide molecules are dominated by through‐space transport. Bulk spectroscopic characterization and density functional theory calculations further reveal that extended amides have a smaller HOMO–LUMO gap and larger transmission values compared to folded amides, consistent with single‐molecule electronic experiments. Overall, this work shows that 3D molecular conformations significantly influence the electronic properties of single‐molecule junctions.

## Graphical abstract

Aromatic foldamers with a trans configuration show enhanced conductance compared to shorter foldamers with a cis configuration due to efficient through‐bond electron tunneling.

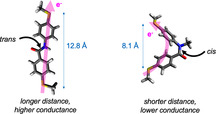

## 1 Introduction

Electron transport in synthetic organic molecules and biomolecules can occur by single‐step (coherent) tunneling, multistep (incoherent) hopping, resonant tunneling, or flickering resonant tunneling.^[^ ^1^, ^2^, ^3^ ^]^ Prior work has shown that nonresonant tunneling is the dominant mechanism for nanoscale charge transport in small molecules,^[^ ^4^, ^5^, ^6^, ^7^, ^8^, ^9^, ^10^, ^11^, ^12^ ^]^ wherein conductance decays exponentially with molecular length or junction displacement. Tunneling distance is a key parameter governing electron tunneling currents,^[^ ^13^ ^]^ but additional factors such as backbone rigidity,^[^ ^14^ ^]^ 3D folded molecular conformations,^[^ ^15^ ^]^ and intramolecular tunneling pathways (through‐bond or through‐space transport)^[^ ^16^, ^17^, ^18^, ^19^, ^20^ ^]^ significantly influence electron tunneling currents. Although recent work has focused on characterizing electron transport in π‐conjugated organic molecules,^[^ ^21^ ^]^ we lack a complete understanding of the role of intramolecular interactions on the electron transport properties of nonconjugated organic molecules and oligomers.

In nature, intramolecular interactions drive the formation of well‐defined 3D folded molecular structures, which in turn impact chemical and biological function.^[^ ^22^ ^]^ Electron transport in peptides^[^ ^15^ ^]^ and nonconjugated organic molecules with heteroatoms^[^ ^23^, ^24^, ^25^ ^]^ depends on molecular composition and noncovalent intramolecular interactions that give rise to secondary structure.^[^ ^26^ ^]^ Recent work has shown that the electronic properties of peptides critically depend on the conformational flexibility of the polyamide backbones, with a high‐conductance state arising due to a folded structure (3_10_ helix or beta turn) and a low‐conductance state occurring for extended peptide structures.^[^ ^15^ ^]^ In recent years, a class of synthetic organic molecules known as foldamers has been developed to understand the role of noncovalent interactions in governing structure‐function relationships.^[^ ^27^, ^28^, ^29^, ^30^, ^31^, ^32^ ^]^ Different classes of foldamers have been used to mimic proteins^[^ ^33^, ^34^, ^35^, ^36^, ^37^, ^38^, ^39^ ^]^ and to achieve desired functional properties such as photoinduced charge transfer and enhanced electron transport.^[^ ^40^, ^41^, ^42^, ^43^ ^]^ In addition, the electron transport properties of π‐conjugated foldamers have been studied at the molecular scale.^[^ ^23^, ^44^, ^45^, ^46^, ^47^ ^]^ Recent work has focused on understanding the molecular electronic properties of various foldamers containing quinoline,^[^ ^40^, ^41^ ^]^ donor‐bridge‐acceptor motifs,^[^ ^42^, ^43^ ^]^ and *ortho*‐phenylene groups.^[^ ^44^, ^45^ ^]^ Prior studies^[^ ^44^, ^45^ ^]^ on foldamers have focused on understanding dynamic structural changes and electronic switching capabilities as steps toward using foldamers in flexible electronics, but foldamers can also be envisioned as model systems for understanding electron transport in biological molecules. Single‐molecule electronic characterization of foldamers containing amide bonds, which resemble peptides or peptoids, can open exciting avenues for understanding fundamental biological electron transport. Despite recent progress,^[^ ^23^ ^]^ however, the single‐molecule electronic properties of foldamers with amide bonds are not yet fully understood.

**Scheme 1:**
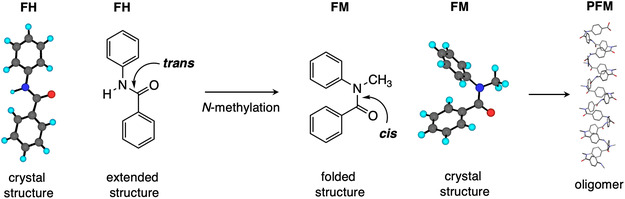
Benzanilide derivative **FH** (foldamer monomer unit with a hydrogen atom on the amide) and its *N*‐methylated compound **FM** (foldamer monomer unit with a methyl group on the amide). The extended secondary amide can be converted to folded *N*‐methylated tertiary amide via *N*‐methylation. Upon oligomerization, the folded *N*‐methylated tertiary amide forms repeats of *N*‐alkylbenzamide (**PFM**), which is known to exhibit dynamic helical properties.^[^ ^20^ ^]^

Prior work reported the synthesis and characterization of foldamers^[^ ^46^ ^]^ with dynamic helical conformations^[^ ^48^ ^]^ based on *N*‐alkylated aromatic amides.^[^ ^20^ ^]^ Secondary amides such as benzamide exist in the *trans*‐amide form, whereas their *N*‐alkylated compound exists in the *cis*‐amide form. These aromatic secondary and tertiary amides are reliable building blocks for preparing longer, extended, and folded molecules, respectively (**Scheme** **1**).^[^ ^20^ ^]^ Foldamers allow for precise characterization of electron transport behavior by providing the ability to link sequence‐defined structure to electronic properties.^[^ ^49^, ^50^ ^]^ However, the effect of structural features or substitutions such as *N*‐methylation on the electronic properties of foldamers is not yet fully understood. From this view, understanding electron transport in foldamers with heteroatom‐containing monomers could provide new insights into the electronic properties of organic molecules with precise secondary structures. Moreover, the ability to design and control the 3D conformation of foldamers provides opportunities for tuning the molecular‐scale electronic properties of organic molecules.

In this work, we report the synthesis and characterization of benzanilide derivative **FH** (foldamer monomer unit with a hydrogen atom on the amide) and its *N*‐methylated compound **FM** (foldamer monomer unit with a methyl group on the amide). Single‐molecule electronic experiments show that **FH** exhibits ≈4x enhancement in molecular conductance compared to **FM** despite a longer molecular contour length. The two aromatic amides differ significantly in terms of molecular conformation and electron tunneling pathways. Bulk spectroscopic experiments and density functional theory (DFT) calculations are used to rationalize results from single‐molecule experiments. Overall, our work shows that molecular composition, conformation, and tunneling pathways significantly influence electron tunneling currents in foldamer molecular junctions.

## 2 Results and Discussion

### 2.1 Chemical Synthesis and Characterization

**Figure 1:**
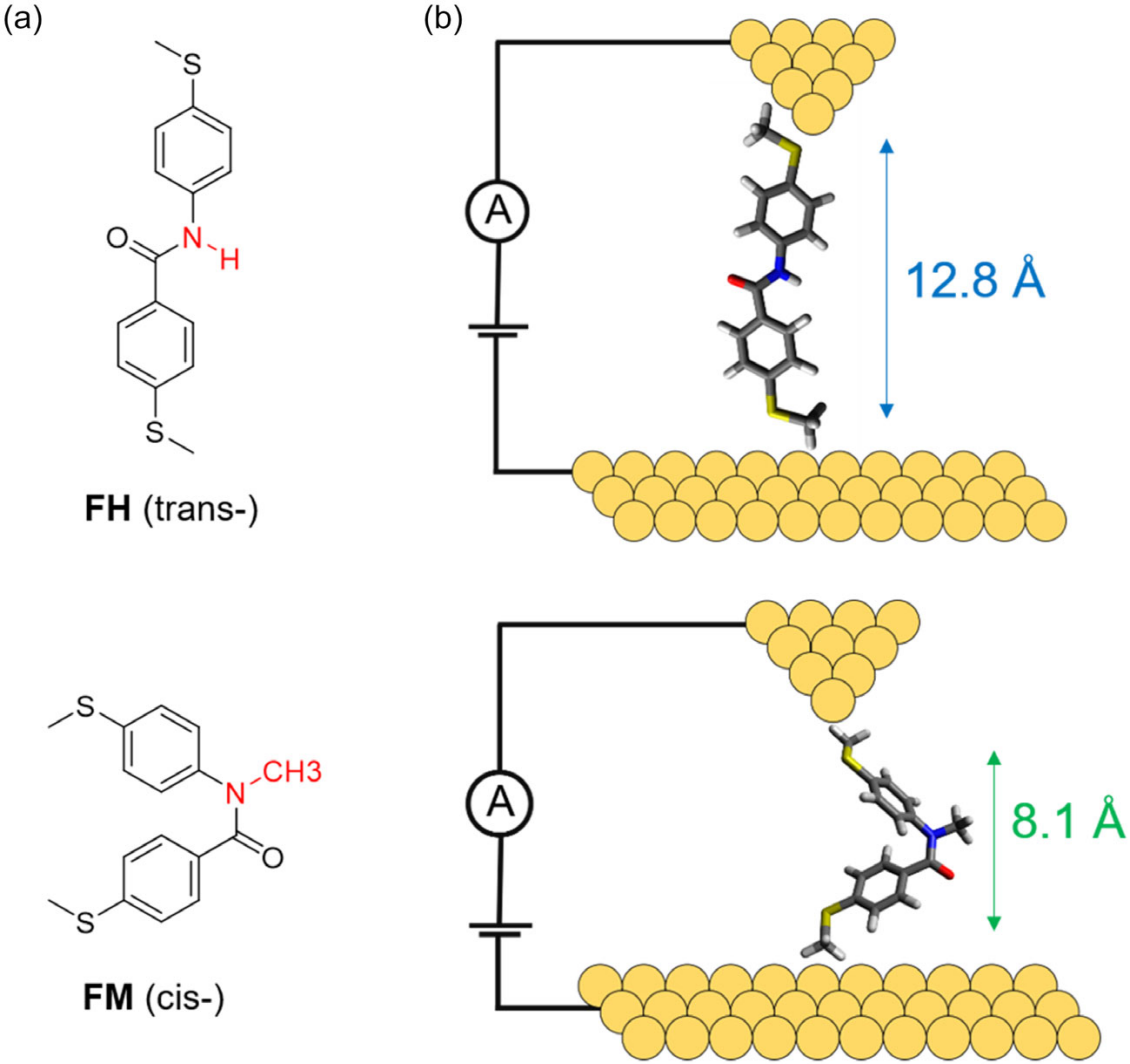
Schematic of experimental setup and foldamer monomer units studied in this work. a) Chemical structures of benzanilide derivative **FH** and *N*‐methylbenzanilide derivative **FM**. b) Schematic of a single‐molecule junction containing **FH** and **FM**.

**Figure 2:**
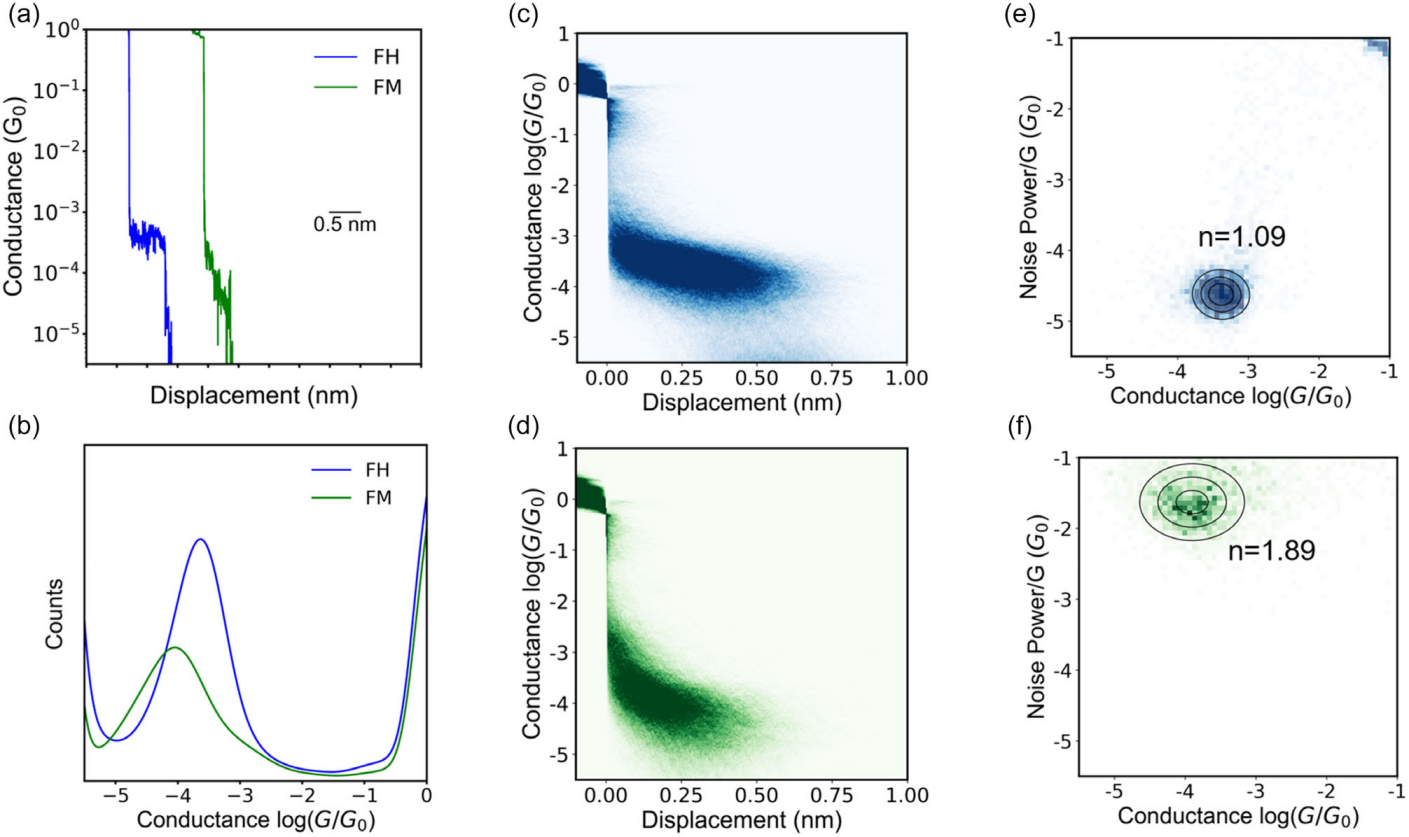
STM‐BJ measurements for benzanilide derivative **FH** and *N*‐methylbenzanilide derivative **FM**. a) Characteristic single‐molecule traces for **FH** and **FM**. b) 1D conductance histogram for **FH** and **FM**. c) 2D conductance histogram for **FH**. d) 2D conductance histogram for **FM**. e) Flicker noise analysis for **FH**, indicating through‐bond mediated electron transport. f) Flicker noise analysis for **FM**, indicating through‐space mediated electron transport. All data were obtained using 0.1 mM concentrations of **FH** and **FM** in 1,2,4‐trichlorobenzene (TCB) solvent at 250 mV applied bias across ensembles of at least 5000 single molecules.

Benzanilide derivative **FH** bearing a secondary amide bond was synthesized via acyl chloride activation and amidation (Figure 1 and Sections S1–S2, Supporting Information). *N*‐Methylbenzanilide derivative **FM** was obtained through *N*‐methylation of **FH** (Figure 1 and Sections S1–S2, Supporting Information). Two terminal methyl sulfide (—SCH_3_) anchor groups were introduced in both molecules to facilitate linkage to gold metal electrodes (**Figure** **1**a). Synthesized molecules were characterized using ^1^H and ^13^C nuclear magnetic resonance (NMR) spectroscopies and mass spectrometry (Figure 2–6, Supporting Information). Prior work showed that benzanilide adopts a *trans*‐conformation, whereas *N*‐methylbenzanilide prefers a *cis*‐conformation, due to differences in electronic and steric effects.^[^ ^51^ ^]^

Crystallographic analysis and DFT calculations were used to characterize the conformations of **FH** and **FM**. A *trans* conformation was observed for **FH** in the crystal structure (Figure 7, Supporting Information). However, **FM** is an oily liquid, which precluded obtaining a crystal structure of this compound. The structure of **FM** was determined by inserting methyl sulfide (‐SCH_3_) groups into the crystal structure of *N*‐methylbenzanilide, followed by geometry optimization using DFT calculations. The DFT‐optimized structures (Figure 8, Supporting Information) suggest that **FM** adopts a *cis* conformation with an S–S distance of 8.1 Å, whereas **FH** maintains a *trans* conformation with an S–S distance of 12.8 Å.

Results from UV–visible spectroscopy experiments show that **FH** exhibits a distinct absorption peak at 291 nm, whereas **FM** shows an absorption peak at 266 nm (Figure 9, Supporting Information). The longer wavelength absorption of **FH** compared to **FM** suggests that **FH** has a larger conjugation length and smaller HOMO–LUMO (highest occupied molecular orbital–lowest unoccupied molecular orbital) gap compared to **FM.** Overall, these results indicate that the two aromatic amide derivatives have stark differences between their molecular composition, conformation, and conjugation length (Figure 1a).

### 2.2 Single Molecule Electronic Measurements

Single‐molecule electronic experiments were used to characterize the electron transport properties of **FH** and **FM**. The foldamer monomer units were characterized using the scanning tunneling microscope‐break junction (STM‐BJ) technique (Figure 1b), as previously described.^[^ ^14^, ^15^, ^52^, ^53^, ^54^, ^55^, ^56^ ^]^ The benzanilide derivative **FH** and *N*‐methylbenzanilide derivative **FM** both contain terminal methyl sulfide (—SCH_3_) groups that readily bind to gold,^[^ ^57^ ^]^ thereby providing robust electrical contacts to metal electrodes in STM‐BJ experiments. Results from STM‐BJ characterization revealed stark differences in the electron transport behavior between **FH** and **FM**, with **FH** exhibiting a well‐defined conductance plateau compared to **FM**, as shown in characteristic single‐molecule conductance traces (**Figure** **2**a and Figure 10, Supporting Information).

One‐dimensional and two‐dimensional molecular conductance histograms were generated for **FH** and **FM** across large ensembles of >5000 single molecules. Our results reveal an average molecular conductance value of ≈10^‐3.7^ *G*_0_ for **FH** compared to ≈10^‐4.1^ *G*_0_ for **FM** (Figure 2b,c,d). The conductance features observed for FH and FM are consistent across a range of bias (Figure 11, Supporting Information), indicating that our molecules do not undergo conformational transitions under an applied bias or in the presence of a mechanical force, unlike prior reports where bias‐induced effects such as tautomerization^[^ ^58^ ^]^ and mechanical force resulted in *cis‐trans* isomerization.^[^ ^59^ ^]^ The foldamers studied in this work differ from previously reported systems containing thioanisole^[^ ^59^ ^]^ because they feature a C=O group instead of an N—H or N—Me group. Prior work has reported that the rotational barriers around C—N bonds in molecular systems^[^ ^60^ ^]^ similar to the amide foldamers in this work are ≈54–80 kJ mol^−1^, which is significantly larger than the rotational barrier around the central C—C bond in a molecule with two phenyl rings separated by a diketone^[^ ^59^ ^]^ (15–45 kJ mol^−1^), presumably due to the partial double‐bond character arising from C—N conjugation. Results from STM‐BJ experiments further show that the average displacements of **FH** and **FM** junctions are 1.06 and 0.82 nm, respectively, after accounting for the snap‐back^[^ ^61^ ^]^ distance arising from atomic rearrangements upon gold nanowire rupture (Figure 12, Supporting Information). The molecular displacements at junction breakage for **FH** and **FM** are consistent with end‐to‐end molecular contour lengths determined from DFT calculations. In the absence of intramolecular interactions, **FM** is expected to show a larger conductance value due to its shorter molecular contour length, assuming that tunneling is the dominant transport mechanism. Nevertheless, electron tunneling currents can be significantly influenced by several additional factors, such as molecular composition, 3D molecular conformation, and tunneling pathways (through‐bond versus through‐space electron transport).

**FH** and **FM** differ in chemical structure at only one position by the presence of either a hydrogen atom or a methyl group on the amide nitrogen atom. However, crystallographic analysis and DFT calculations show significant differences in molecular conformation between these molecules, with **FH** adopting a *trans*‐conformation and **FM** adopting a *cis*‐conformation (Figure 7–8, Supporting Information). Additionally, molecular orbital analysis (*vide infra* *)* and prediction of HOMO–LUMO gaps for FH and FM indicate that the introduction of a methyl group on the backbone nitrogen in place of the hydrogen leads to changes in electronic properties.

To understand the electron tunneling pathways for **FH** and **FM**, we performed flicker noise analysis^[^ ^58^ ^]^ to differentiate between through‐bond and through‐space electron transport.^[^ ^62^ ^]^ Prior work has shown that the conductance fluctuations (quantified by analyzing the power spectral density (PSD) of conductance noise) exhibit a power law dependance on the mean conductance *G* values, depending on through‐space and through‐bond transport characteristics.^[^ ^63^, ^64^ ^]^ Conductance noise is quantified by numerically integrating the conductance noise PSD between frequencies of 100 and 1000 Hz.^[^ ^54^, ^62^ ^]^ The conductance behavior is then characterized by the scaling exponent *n* of the normalized noise power (noise power/*G* ^n^) versus the average normalized conductance *G/G*_0_, where *G*_0_ is the conductance quantum. A scaling exponent *n* ≈ 2 suggests through‐space transmission, whereas an exponent *n* ≈ 1 corresponds to through‐bond transport.^[^ ^62^, ^63^ ^]^ Our results show that the scaling exponent for **FH** is 1.09 and for **FM** is 1.89, indicating that **FH** exhibits through‐bond mediated electron transport, whereas **FM** exhibits through‐space mediated electron transport (Figure 2e,f and Figure 13, Supporting Information). Based on these results, we posited that the folded structure of **FM** brings the phenyl rings into close proximity, thereby enabling through‐space electron tunneling.^[^ ^65^, ^66^ ^]^

We next performed single‐molecule electronic experiments for **FH** and **FM** in propylene carbonate (PC) solutions to understand the role of the solvent environment on molecular conductance. Our results show that **FH** has higher conductance compared to **FM** in nonpolar and polar solvents (Figure 14–15, Supporting Information). Interestingly, FH exhibits nearly the same junction formation probability in both polar (PC) and nonpolar solvents (TCB), whereas FM shows a significantly lower junction formation probability in PC as compared to TCB, suggesting that the solvent can influence the electronic properties of foldamers depending on their chemical structure, 3D folding, and electron tunneling pathways (Table 1, Supporting Information). Shorter aromatic amine foldamers exhibit lower conductance values due to through‐space electron transport, whereas longer foldamers appear to show higher conductance values due to through‐bond electron transport. Overall, these results suggest that electron tunneling currents are significantly influenced by molecular composition, 3D conformation, and electron tunneling pathways.

### 2.3 DFT Calculations

**Figure 3:**
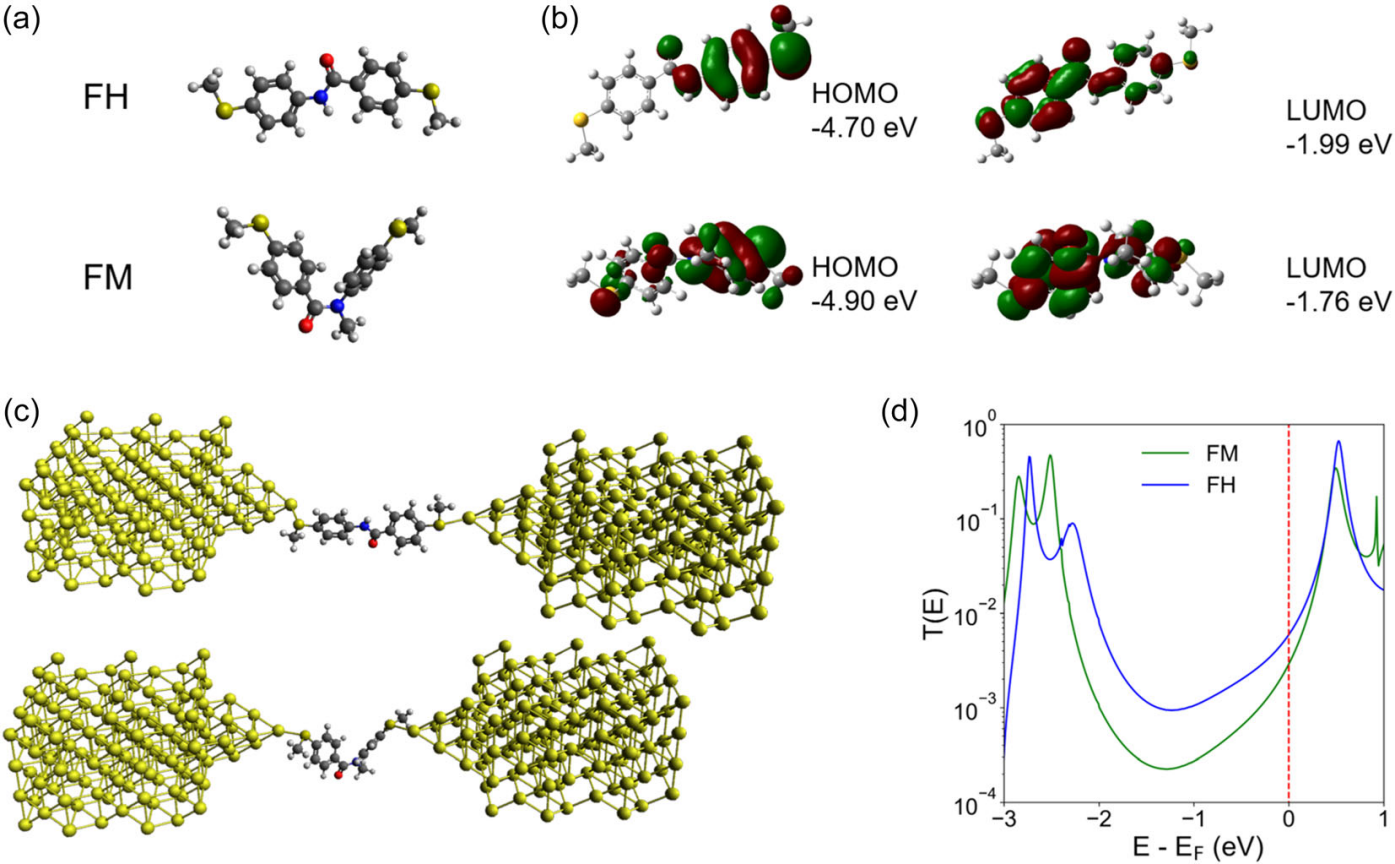
DFT calculations for **FH** and **FM**. a) Geometry optimized structures indicating that **FH** and **FM** adopt a *trans* and *cis* conformation, respectively. b) Molecular orbital isosurfaces for **FH** and **FM**. c) Molecule electrode geometries in nanoscale junctions for NEGF‐DFT calculations. d) Transmission probability values for **FH** and **FM** as a function of energy relative to the Fermi energy level.

To complement experimental results, we performed DFT calculations, molecular orbital visualization, and nonequilibrium Green's function‐density functional theory (NEGF‐DFT) calculations. DFT calculations are performed using B97D/6‐311G(d,p) level of theory to identify the optimized structures of **FH** and **FM** (Figure 3a and Sections S5.1–S5.2, Supporting Information). Our results show that **FH** adopts a *trans* conformation, whereas **FM** adopts a *cis* conformation, consistent with crystallographic analysis. Following geometry optimization using B97D/6‐311G(d,p), single‐point calculations were performed using B97D/STO‐3G to identify the vacant orbitals.

Molecular orbitals for **FH** and **FM** reveal stark differences in terms of orbital overlap and frontier molecular orbital energies (**Figure** **3**b and Table 2, Supporting Information). Molecular orbital analysis indicates that **FH** has a smaller HOMO–LUMO gap compared to **FM**, consistent with bulk spectroscopy experiments. NEGF‐DFT calculations were performed for **FH** and **FM** (Figure 3c). Transmission calculations reveal higher electron transmission for **FH** as compared to **FM** (Figure 3d), qualitatively consistent with single‐molecule electronic experiments. The transmission values of the resonance peaks for **FH** and **FM** are below unity, as the molecules lack full conjugation along their entire backbone. This behavior is not expected to arise due to an Au–S gateway state,^[^ ^67^, ^68^ ^]^ but rather reflects the intrinsic electronic structure of the molecule. Overall, DFT calculations corroborate the results from single‐molecule electronic experiments and bulk spectroscopy.

## 3 Conclusions

In this work, the electronic properties of foldamer monomer units, including the benzanilide derivative **FH** and the *N*‐methylbenzanilide derivative **FM**, are characterized using a combination of single‐molecule electronic experiments, bulk spectroscopy, and DFT calculations. Although these molecules exhibit only a small difference in structure, with **FH** containing a hydrogen atom and **FM** containing a methyl group on the amide nitrogen atom, they adopt starkly different molecular conformations, with **FH** adopting a *trans* conformation and **FM** a *cis* conformation. Single‐molecule experiments reveal that **FH** shows ≈4x enhancement in average molecular conductance compared to **FM**, despite its longer contour length. In addition, **FH** and **FM** exhibit different electron transport mechanisms, with **FH** showing through‐bond electron transport and **FM** exhibiting through‐space electron transport. Results from UV‐visible spectroscopy are consistent with a longer conjugation length and smaller energy gap for **FH** compared to **FM**. DFT calculations and molecular orbital analysis further show that **FH** has a smaller HOMO–LUMO gap compared to **FM**. NEGF‐DFT calculations reveal **FH** exhibits higher transmission values than **FM**, qualitatively consistent with results from single‐molecule break junction experiments.

This work suggests that molecular distance is not the sole factor determining electron tunneling currents in molecular junctions. Additional properties such as molecular substitution patterns, 3D folding, molecular conformation, and electron tunneling pathways significantly influence electron tunneling currents. From this view, elucidating structure‐function relationships for flexible molecules such as foldamers offers the potential to control the electronic behavior of organic molecules. Finally, we note that the extended secondary amide studied in this work is chemically analogous to a peptide backbone,^[^ ^15^ ^]^ whereas the folded tertiary amide more closely resembles a peptoid backbone.^[^ ^69^, ^70^ ^]^ The foldamer systems in this work demonstrate the possibility of observing distinct electron transport fingerprints for peptides and peptoids, as evidenced by FH exhibiting higher conductance than FM. Overall, our work could open new avenues for using foldamers as model organic systems to mimic the structural behavior of biomolecules (e.g., peptides and peptoids) for electronic applications.

## Conflict of Interest

The authors declare no conflict of interest.

## Author Contributions

**Rajarshi Samajdar**, **Xiaolin Liu**, **Aya Tanatani**, and **Charles M. Schroeder** conceived this study. **Rajarshi Samajdar** performed single‐molecule experiments and data analysis. **Kazusa Kuyama** performed chemical synthesis. **Yui Kidokoro** and **Fumi Takeda** performed UV spectroscopy. **Fumi Takeda** performed NMR characterization. **Masatoshi Kawahata** and **Kazusa Kuyama** performed X‐ray crystallographic analysis at Spring‐8. **Iwao Okamoto** performed DFT calculations. **Rajarshi Samajdar** performed NEGF‐DFT calculations. **Aya Tanatani**, **Jeffrey S. Moore**, and **Charles M. Schroeder** supervised the research. The manuscript was written by **Rajarshi Samajdar**, **Aya Tanatani**, and **Charles M. Schroeder** with contributions from all authors.

## Supporting information

Supplementary Material

## Data Availability

The data that support the findings of this study are available from the corresponding author upon reasonable request.
