# Supplementary material for: Aromatic Amide Foldamers Show Conformation‐Dependent Electronic Properties
Source: Chemphyschem. 2025 Nov 8;26(24):e202500672. doi: 10.1002/cphc.202500672 (PMC12710157; doi:10.1002/cphc.202500672)
Supplement: Supplementary file 1 — Supplementary Material [file CPHC-26-e202500672-s001.pdf]

Supplementary Information for:

Aromatic amide foldamers show conformation-dependent electronic properties

Rajarshi Samajdar[1,2], Xiaolin Liu[3], Kazusa Kuyama[4], Yui Kidokoro[4], Fumi Takeda[4], Iwao Okamoto[5], Masatoshi Kawahata[5], Kosuke Katagiri[6], Jeffrey S. Moore[2,3,7], Aya Tanatani[4*], Charles M. Schroeder[1,2,3,7*†]

[1]Department of Chemical and Biomolecular Engineering, University of Illinois at Urbana-Champaign, Urbana, Illinois, 61801
[2]Beckman Institute for Advanced Science and Technology, University of Illinois at Urbana-Champaign, Urbana, Illinois, 61801
[3]Department of Chemistry, University of Illinois at Urbana-Champaign, Urbana, Illinois, 61801
[4]Department of Chemistry, Faculty of Science, Ochanomizu University, 2-1-1 Otsuka, Bunkyo-ku, Tokyo 112-8610, Japan
[5]Faculty of Pharmaceutical Science, Showa Pharmaceutical University, 3-2-1 Higashitamagawagakuen, Machida, Tokyo 194-8543, Japan
[6]Department of Chemistry of Functional Molecules, Faculty of Science and Engineering, Konan University, 8-9-1 Okamoto, Higashinada, Kobe, Hyogo 658-8501, Japan
[7]Department of Materials Science and Engineering, University of Illinois at Urbana-Champaign, Urbana, Illinois, 61801

*Corresponding authors: Aya Tanatani, email: tanatani.aya@ocha.ac.jp; Charles M. Schroeder, email: cschroeder@princeton.edu
†Present address: Department of Chemical and Biological Engineering, Princeton University, 41 Olden Street, Princeton, NJ 08544

Table of Contents

S1. General methods…………………………………………………………..………... 3

S2. Chemical synthesis and characterization ………………………………………6

S3. Bulk spectroscopy ……………………………………………………..…....… 11

S4. Single molecule electronic experiments ……………….………………………12

S5. Density functional theory calculations……………………………..……………17

S6. References……………………………..………………………………………… 21

S1. General methods

Chemicals

4-(Methylthio)benzoic acid, dimethylformamide (DMF), dry CH_2Cl_2, and dry pyridine were purchased from Wako Pure Chemical Industries. Oxalyl chloride and 4-(methylthio)aniline were obtained from Tokyo Kasei Kogyo Co. and Sigma-Aldrich Chemical Co., respectively. Methyl iodide and sodium hydride (55%) were purchased from Kanto Kagaku Co. Silica Gel 60 N (spherical, neutral) and Kieselgel 60 (230–400 mesh) for column chromatography were purchased from Kanto Kagaku Co., Inc. and Merck, respectively.

Chemical characterization

NMR. ^1H and ^{13}C NMR spectra were recorded on a Bruker Avance 600 spectrometer. NMR spectra are referenced to residual DMSO (δ = 2.50 ppm, ^1H; 39.52 ppm, ^{13}C) or chloroform (δ = 7.26 ppm, ^1H; 77.16 ppm, ^{13}C).

Mass spectrometry. Electrospray Ionization (ESI) spectra were performed on a Waters Synapt G2-Si ESI mass spectrometer.

UV-visible spectroscopy

UV spectra were recorded with a JASCO V-650 spectrophotometer using a 1 cm quartz cell.

X-ray crystallography

In the X-ray crystallographic analysis, a single crystal was immersed in Paratone-N oil and placed in a cold nitrogen stream at 100 K. Data were collected using a diffractometer with an EIGER 4M PAD detector (SPring-8 BL26B1, synchrotron: λ = 0.800 Å). Absorption correction was performed by a multi-scan method implemented in XDS. Structure solution and refinement were performed by using SHELXT-2018/2 and SHELXL-2019/3. CCDC 2432956

Single molecule conductance measurements

Single-molecule conductance measurements were performed using a custom-built scanning tunneling microscope break junction (STM-BJ), as previously reported.[1–3] Gold substrates were prepared by evaporating 100 nm of gold onto polished Ted Pella AFM specimen disks with an e-beam evaporator. STM tips were prepared with 0.25 mm Au wire (99.998%, Alfa Aesar). Measurements were performed in 1,2,4-tricholorbenzene (TCB) and propylene carbonate (PC) solvents (where noted). For the measurements carried out in PC solvent, the STM tips were coated with Apiezon wax to minimize the exposed area to polar solvents and to reduce the non-Faradaic current[4]. During experiments, the STM tip is controlled by a piezoelectric micro-positioner to repeatedly form and break molecular junctions, and the current was recorded and analyzed during this process. A variable-gain low noise current amplifier (DLPCA-200 from Artisan

Technology Group) was used to accurately convert current to voltage for data processing. Conductance histograms (determined from > 5000 individual molecules per experiment) are generated for all molecules without data selection. The 1D conductance histograms were generated on a logarithmic scale [log(G/G_0)] from −5.5 to 0 with a bin size of 0.01 and subsequently smoothed using a 1D Gaussian filter with a standard deviation of 20 bins.

Flicker noise analysis

Flicker noise analysis was performed to differentiate between through-bond and through-space electron transport modes[5]. To perform this analysis, the conductance fluctuations were experimentally determined while holding molecular junctions at a fixed tip-to-substrate separation. In these experiments, a ramp start displacement of 2.5 nm, a holding length of 3.5 nm, a final pull length of 3 nm, and a cap length of 0.1 nm were used, accounting for a total displacement of 9.2 nm. The pulling rate was 20 nm/s, and the data acquisition rate was 40 kHz. The measurement was repeated for > 15,000 molecules for the molecules studied in this work. Only junctions that survive the entire holding sessions were considered for further analysis (examples of sample holding traces are included in **Supplementary Figure 13a,c**). The discrete Fourier transformation was applied to the data of the 'holding' section (and squared) to obtain the noise power spectral density (PSD) of each trace (examples of PSD of traces are included in **Supplementary Figure 13b,d)**. The noise power was quantified by numerically integrating the PSD from 100 Hz to 1 kHz and normalized by the average conductance of the corresponding trace. The relationship between the normalized noise power (PSD/G^n) and the average conductance (G) was calculated by bivariate normal distribution fitting to the 2D histograms, where n is the scaling exponent of the average conductance. The fitting parameters include amplitude, peak centers, standard deviations, correlation coefficient, and background offset. The noise power was systematically optimized from 1.0 to 2.0 to determine the exponent of the conductance dependence that minimized the correlation coefficient in the 2D Gaussian fit. A scaling factor of $n \approx 1$ indicates through-bond conductance, whereas a scaling factor of $n \approx 2$ indicates through-space conductance.

Density functional theory (DFT) calculations

DFT calculations were performed using the Gaussian16 suite (Rev C. 01)[6]. The geometry optimization of foldamer H and M were carried out using B97D/6-311G(d,p). For the molecular orbital visualization, optimization was carried out using B97D/6-311G** followed by single point calculation was performed using B97D/STO-3G to figure out the vacant orbitals.

Non-equilibrium Green's function-density functional theory (NEGF-DFT)

NEGF-DFT calculations are performed with a DFT-based non-equilibrium Green's function (NEGF) approach using the TranSiesta and Tbtrans package[7–9]. The electrodes contain 8 layers of 16 gold atoms along with a pyramid of 10 Au atoms. The methyl sulfide (-SCH_3) groups on the molecules were made to interact with the gold electrodes. Geometry relaxation of the molecules was performed using generalized gradient approximation-Perdew-Burke-Ernzerhof (GGA-PBE) functional[10] using the TranSiesta

package[8]. DZ basis sets were used for all the gold atoms. DZP basis sets were used for carbon, hydrogen, oxygen and nitrogen. Electrode calculations were carried out with a $4 \times 4 \times 50$ k-mesh. The geometry relaxation was carried out using a $4 \times 4 \times 1$ k-mesh, which was performed until the forces were < 0.05 eV/Å. After the junction was relaxed, the transport calculations were carried out using the TranSiesta package[7,9] with the same functionals, basis sets, pseudopotential, and k-mesh as the geometry relaxation. Convergence was tested prior to transmission calculations, using a real axis integration internal from -40 eV to infinity[7]; this includes a crossing in the imaginary axis at 2.5 eV, and the γ value is $-10k_{\mathrm{B}}T$. The circle grid consists of 102 Gauss-Legendre points, and 15 Gauss-Fermi points for the tail portion. Tbtrans[9] was used to carry out the NEGF calculations and to obtain electron transmission as a function of energy (relative to the fermi energy level). NEGF calculations were carried out from -3 eV to 1 eV with 0.01 eV energy increments[11].

S2. Chemical synthesis and characterization

FH and **FM** were synthesized according to the scheme shown below.

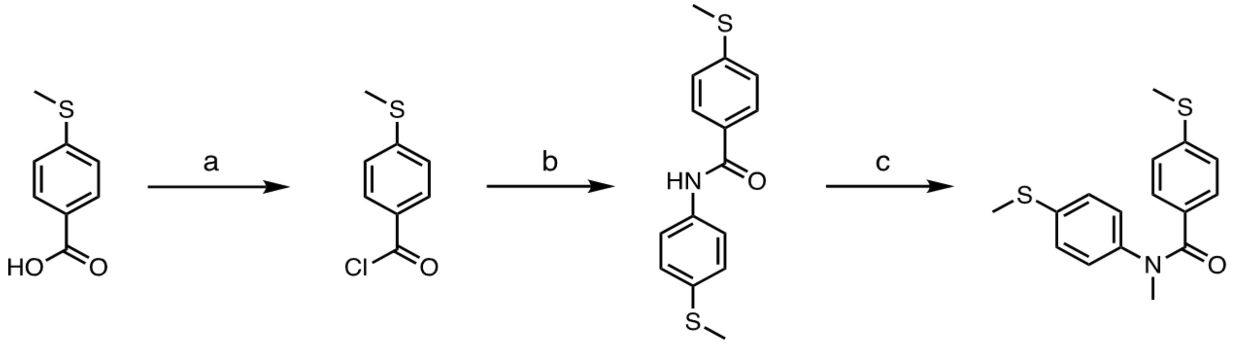

1 FH FM

Supplementary Figure 1: Synthesis of **FH** and **FM**. Reagents and Conditions: (a) oxalyl chloride, DMF, CH_2Cl_2, room temperature. (b) 4-(methylthio) aniline, pyridine, room temperature. (c) NaH, CH_3I, DMF, room temperature.

A solution of oxalyl chloride (314 mg, 2.48 mmol) in dry CH_2Cl_2 (2 mL) was added dropwise to a solution of 4-(methylthio)benzoic acid (213 mg, 1.26 mmol) and DMF (2 drops) in CH_2Cl_2 (3 mL) at 0℃. The mixture was stirred at room temperature for 3 h. The solvent was removed in vacuo to give 1 as a yellow solid (295 mg, quant.).

Compound 1: yellow solids; ^{1}H NMR (600 MHz, $CDCl_3$) δ 8.00 (dt, J = 1.8, 9.0 Hz, 2 H), 7.28 (dt, J = 1.8, 8.4 Hz, 2 H), 2.54 (s, 3 H).

Compound 1 (182 mg, 0.97 mmol) was added to a solution of 4-(methylthio)aniline (72 mg, 0.52 mmol) in dry pyridine (4 mL), and the mixture was stirred at room temperature for 4 h. The reaction mixture was quenched with 2 M hydrochloric acid. The precipitates were collected, and washed with MeOH to give **FH** as pink solids (94 mg, 62%).

FH: pink solids; m.p. 215-216 ˚C; ^{1}H NMR (600 MHz, DMSO-d_6) δ 7.90 (dt, J = 1.8, 8.4 Hz, 2 H), 7.73 (dt, J = 1.8, 8.4 Hz, 2 H), 7.38 (dt, J = 1.8, 8.4 Hz, 2 H), 7.26 (dt, J = 1.8, 9.0 Hz, 2 H), 2.54 (s, 3 H), 2.46 (s, 3 H);^{13}C NMR (150 MHz, DMSO-d_6) δ 164.7, 143.0, 136.6, 132.2, 130.7, 128.1, 126.9, 124.9, 121.0, 15.5, 14.1; HRMS (ESI+) m/z: calcd for $C_{15}H_{15}NOS_2$ $[M+H]^+$ 290.0667; found 290.0677.

A solution of **FH** (44 mg, 0.15 mmol) and methyl iodide (61 mg, 0.43 mmol) in dry DMF (4 mL) was stirred at room temperature for 10 min. Sodium hydride (55%, 14 mg, 0.323 mmol, washed with *n*-hexane twice) in dry DMF (1 mL) was added to the mixture at 0˚C. The reaction mixture was stirred at room temperature for 2 h, and the solvent was removed in vacuo. The residue was poured into CH_2Cl_2, and filtered. The filtrate was poured into water, and extracted with CH_2Cl_2. The organic layer was washed with brine, dried over $MgSO_4$, and filtered. After evaporation, the residue was purified by preparative

thin-layer chromatography (AcOEt/*n*-hexane 1: 3) to give **FM** as colorless oil (43 mg, 92%).

FM : colorless oil; ^{1}H NMR (600 MHz, $CDCl_3$) δ7.22 (dt, *J* = 1.8, 8.4 Hz, 2 H), 7.10 (dt, *J* = 1.8, 8.4 Hz, 2 H), 7.01 (dt, *J* = 1.8, 8.4 Hz, 2 H), 6.95 (dt, *J* = 1.8, 8.4 Hz, 2 H), 3.45 (s, 3 H), 2.44 (s, 3 H), 2.42 (s, 3 H); ^{13}C NMR (150 MHz, $CDCl_3$) δ 170.2, 142.2, 141.3, 137.0, 132.0, 129.6, 127.3, 127.1, 124.9, 38.7, 15.8, 15.1; HRMS (ESI+) m/z: calcd for $C_{16}H_{17}NOS_2$ $[M+H]^+$ 304.0824; found 304.0830.

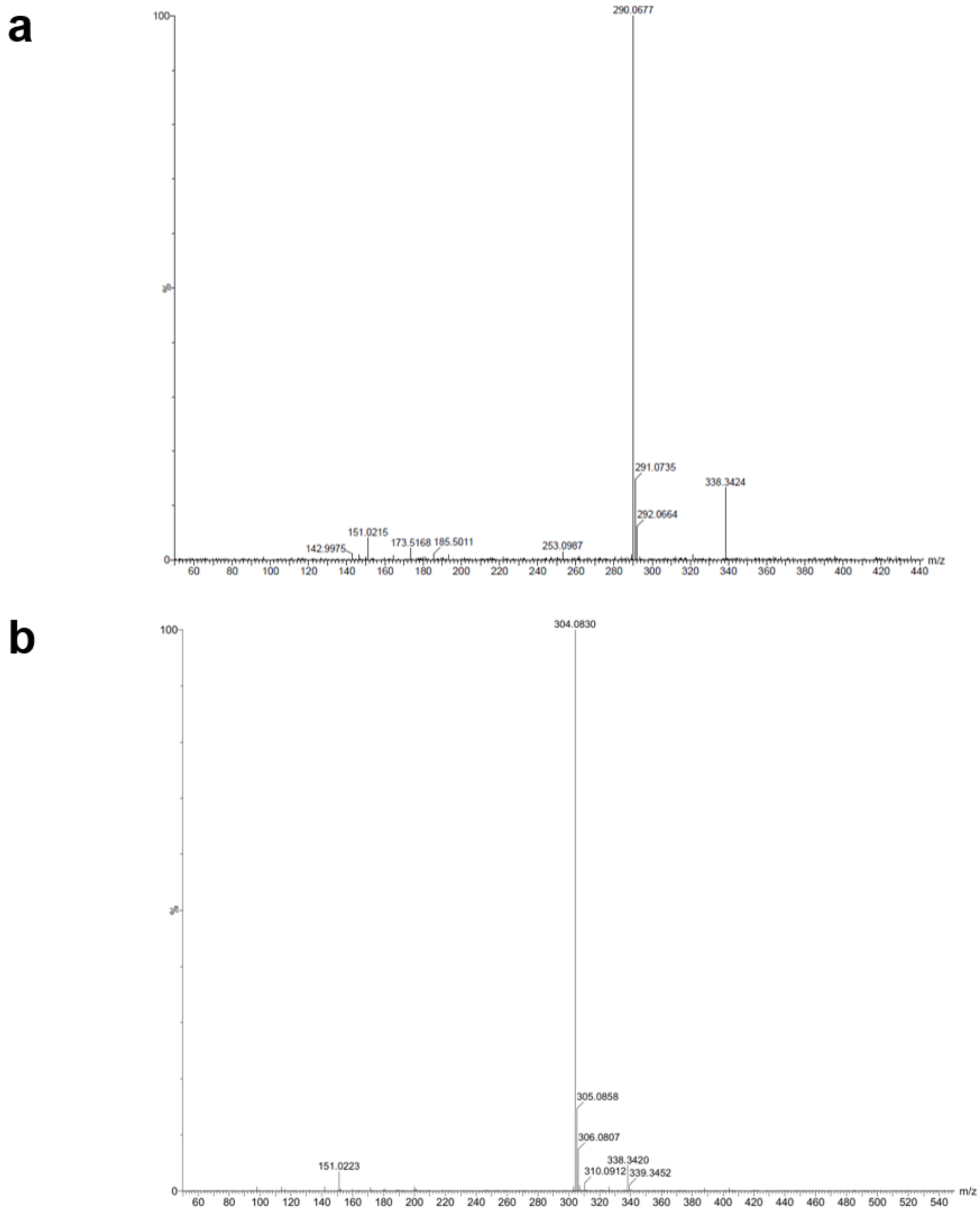

Supplementary Figure 2: ESI+ Mass Spectrometry for **FH** and **FM**. (a) Observed molecular weight for **FH** was 290.0677. (b) Observed molecular weight for **FM** was 304.0830.

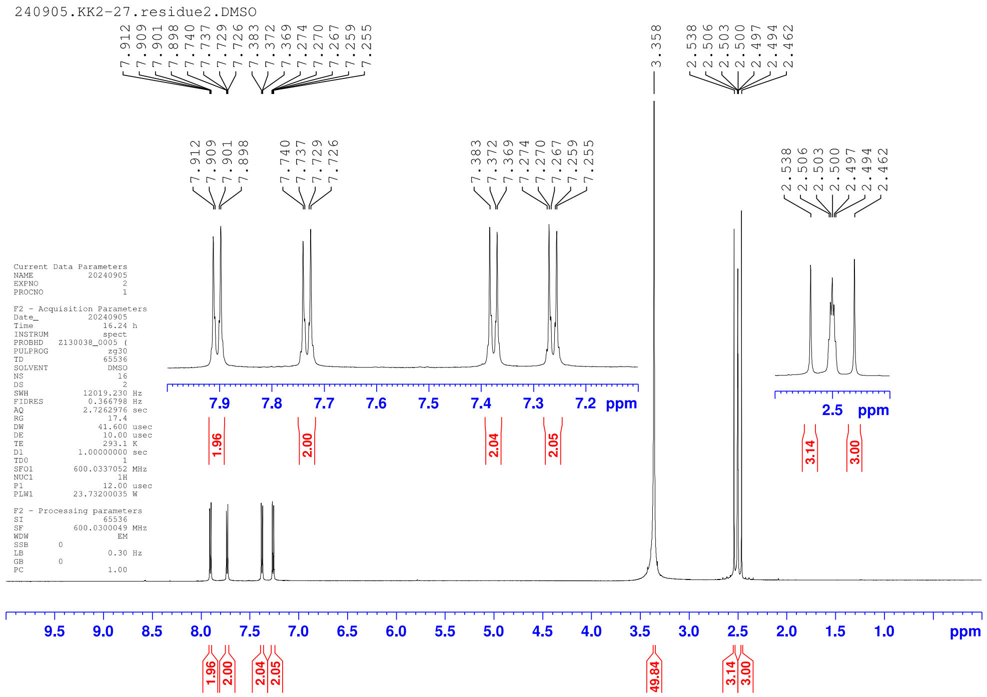

Supplementary Figure 3: ^{1}H-NMR of **FH** (600 MHz, DMSO-d_6)

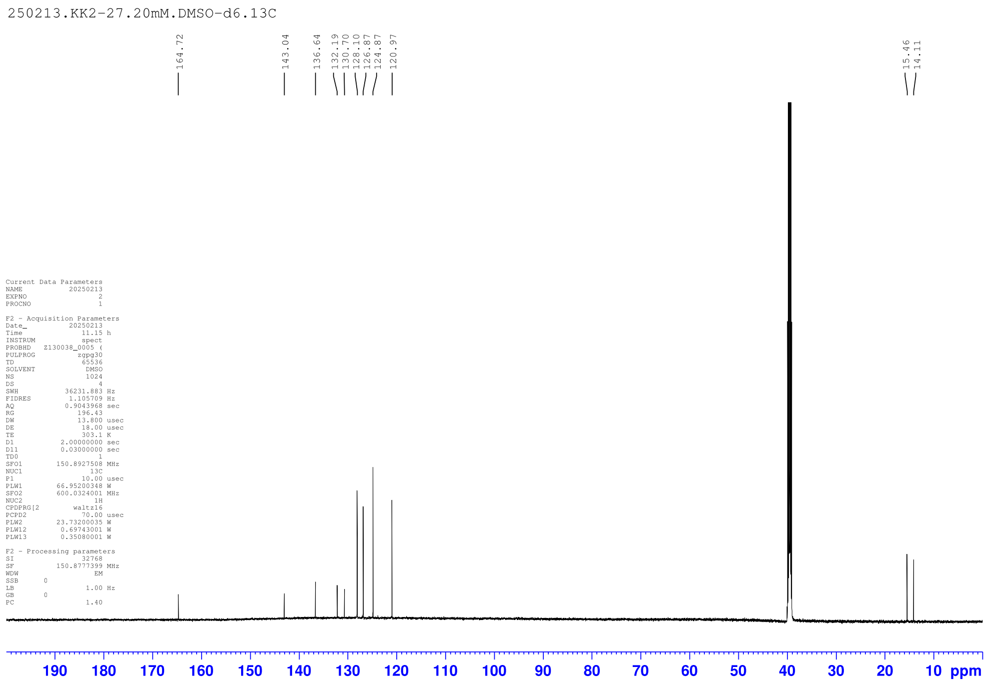

Supplementary Figure 4: ^{13}C-NMR of **FH** (150 MHz, DMSO-d_6)

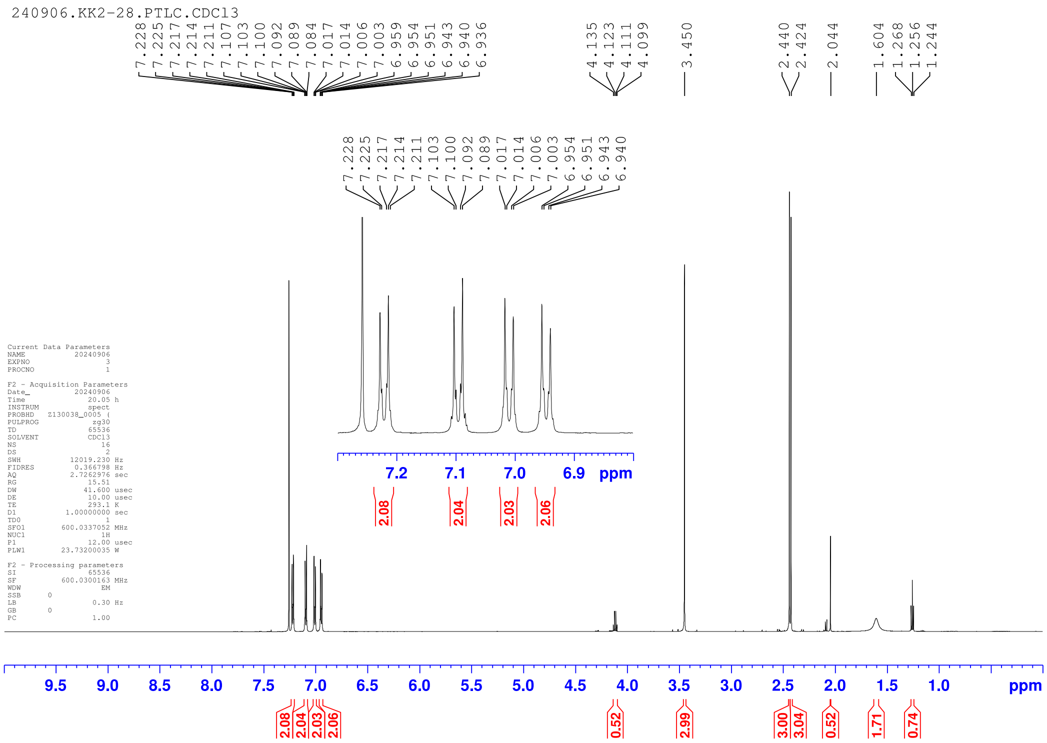

Supplementary Figure 5: ^{1}H-NMR of **FM** (600 MHz, $CDCl_3$)

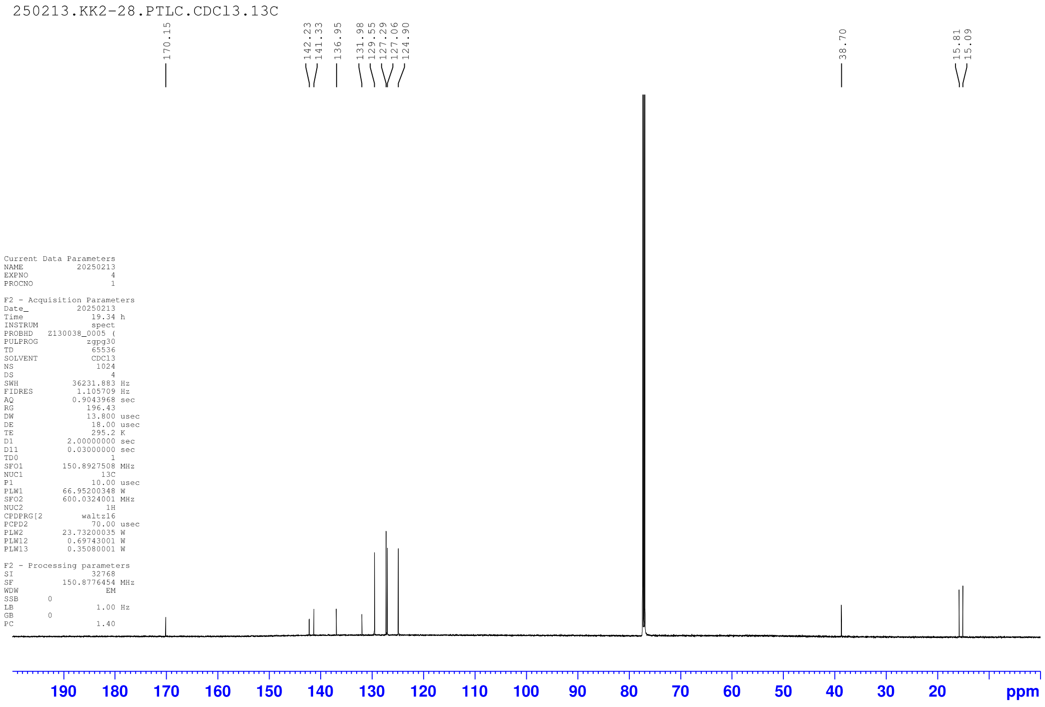

Supplementary Figure 6: ^{13}C-NMR of **FM** (150 MHz, $CDCl_3$)

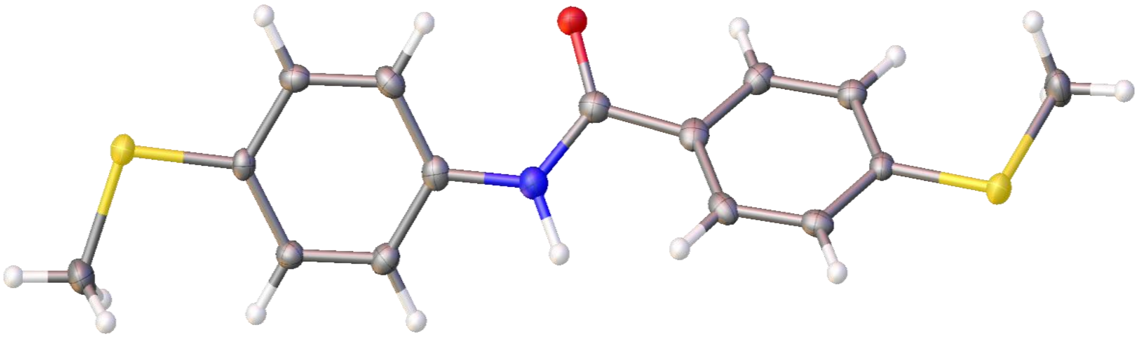

Supplementary Figure 7: Crystal structure of **FH.**

a

b

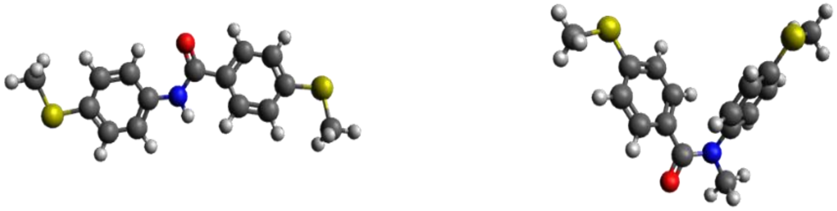

Supplementary Figure 8: Density functional theory (DFT) optimized structures of (a) **FH** and (b) **FM**. **FH** structure was obtained using crystallographic analysis, as depicted in **Supplementary Figure** 7. **FM** structure was obtained by addition of thiomethyl groups on N-methylbenzanilide and optimizing it using DFT.

S3. Bulk spectroscopy

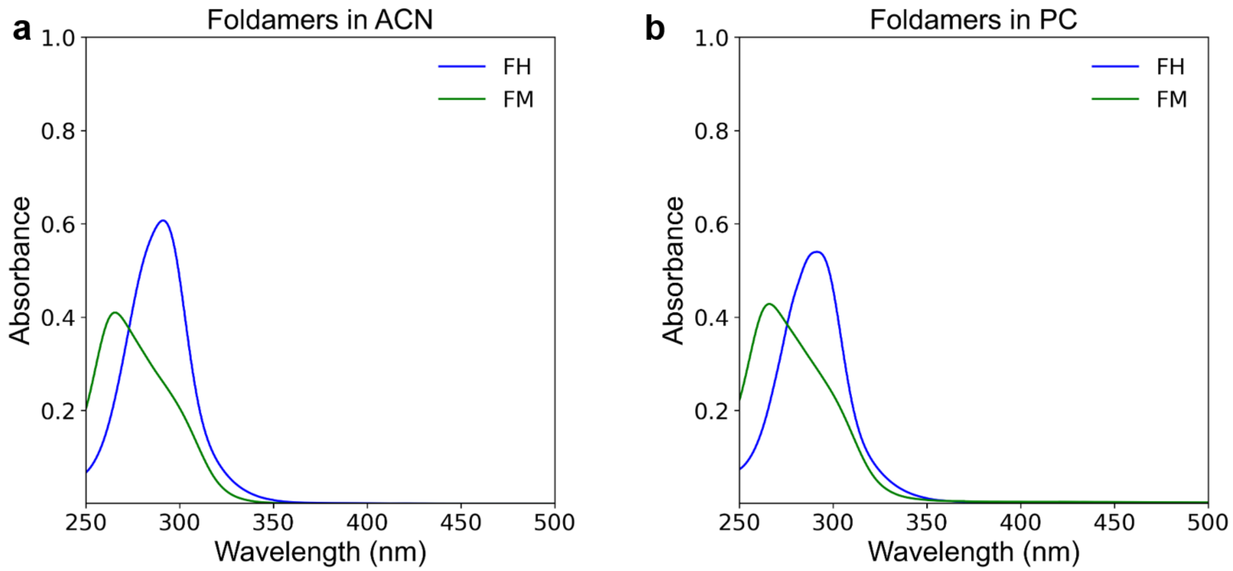

Supplementary Figure 9: UV-visible spectroscopy for foldamer **FH** and foldamer **FM** in (a) acetonitrile and (b) propylene carbonate as solvents. Absorption peak occurs at 291 nm and 266 nm for foldamer **FH** and **FM** respectively. The experiments were carried out with 0.1 mM FH and FM in acetonitrile and propylene carbonate.

S4. Single molecule electronic experiments

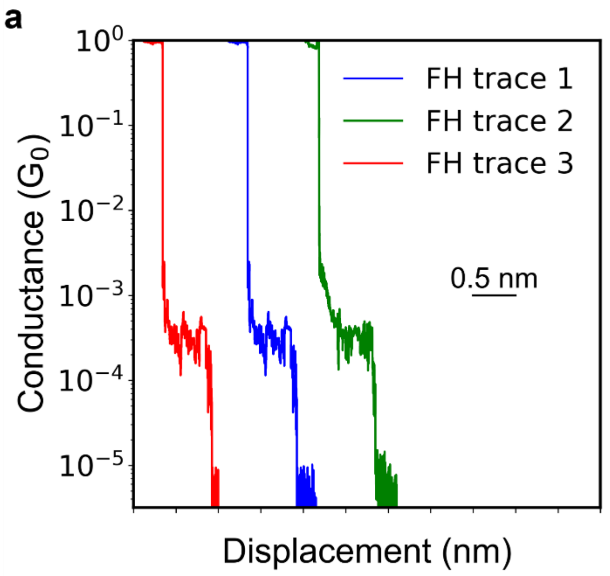

Supplementary Figure 10: Characteristic single molecule traces for FH and FM as observed in molecular scale break junction experiments. The experiments are carried out at 250 mV applied bias using 0.1 mM concentrations of **FH** and **FM**.

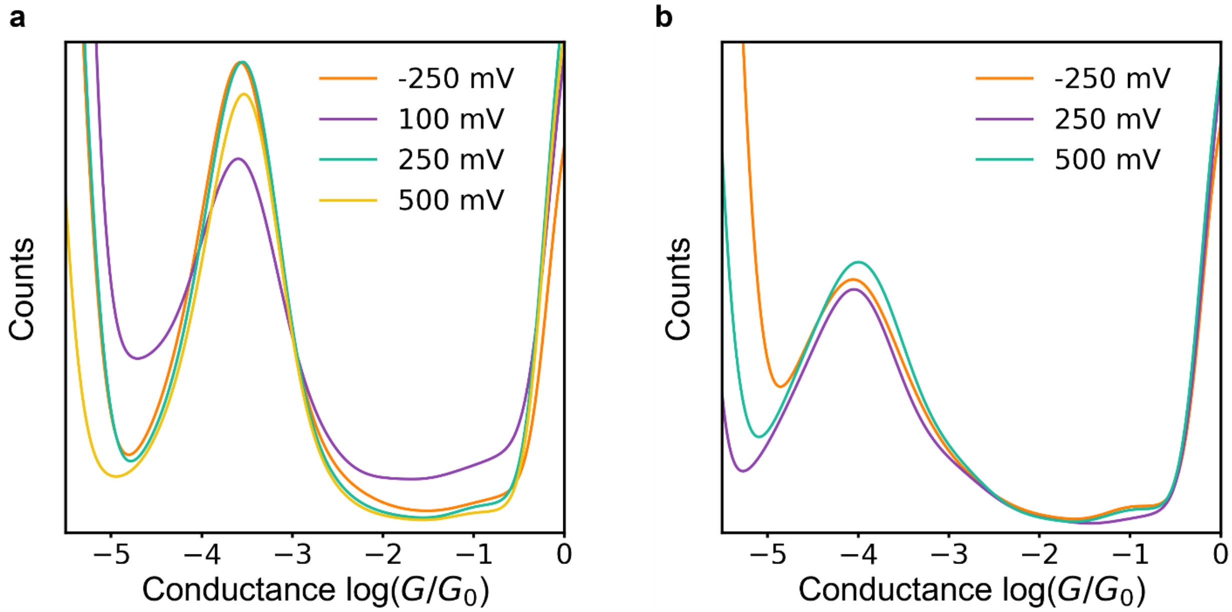

Supplementary Figure 11: Bias dependent molecular scale experiments for (a) **FH and** (b) **FM**. Data were obtained using 0.1 mM concentrations of **FH** and **FM** in 1,2,4-tricholorbenzene (TCB) solvent at varied applied bias across ensembles of at least 5000 single molecules.

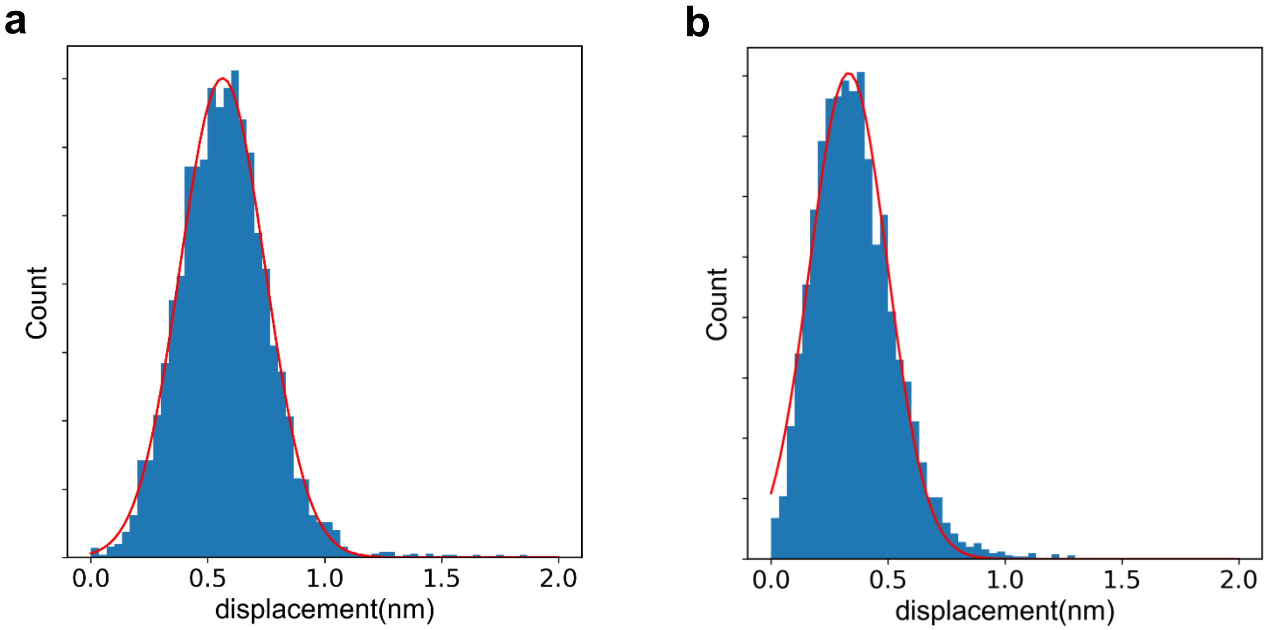

Supplementary Figure 12: Displacement histograms for **FH** and **FM**. (a) Average length of junctions formed by **FH** is 0.56 nm. (b) Average length of junctions formed by **FM** is 0.32 nm. The length of junctions are 1.06 nm and 0.82 nm for **FH** and **FM** after accounting for snapback distance[11]. Data were obtained using 0.1 mM concentrations of **FH** and **FM** in 1,2,4-trichlorobenzene (TCB) solvent at 250 mV applied bias across ensembles of at least 5000 single molecules.

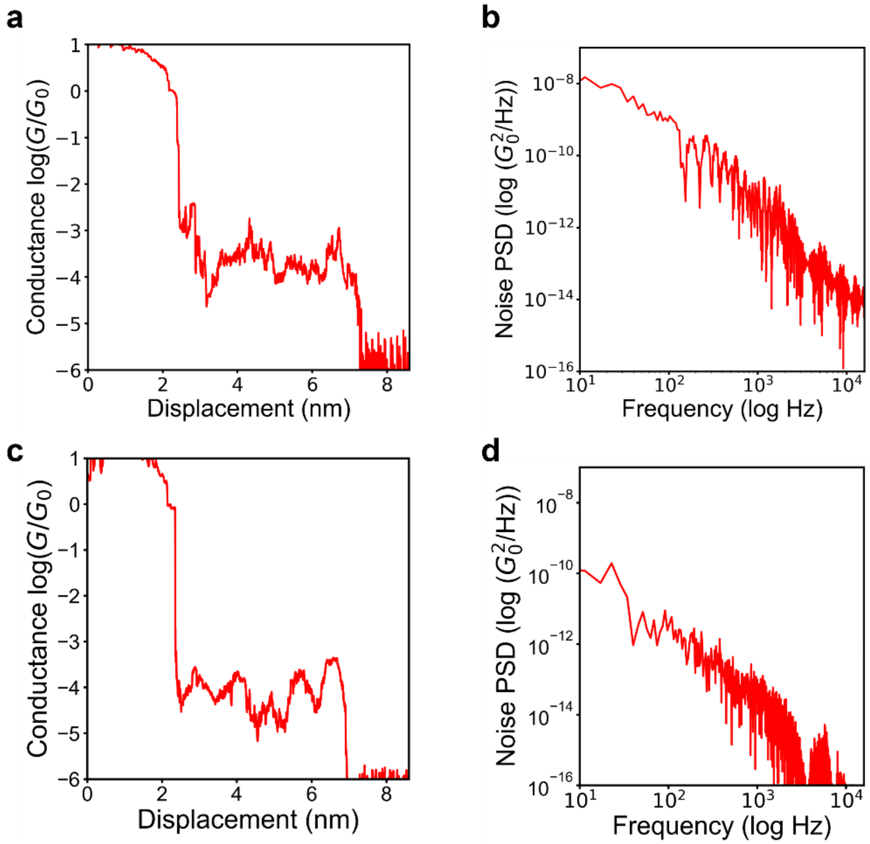

Supplementary Figure 13: Flicker noise analysis for FH and FM. (a) Representative holding-mode conductance traces for **FH**. (b) Characteristic noise power spectral density (PSD) traces for **FH**. (c) Representative holding-mode conductance traces for **FM**. (d) Characteristic noise power spectral density (PSD) traces for **FM.**

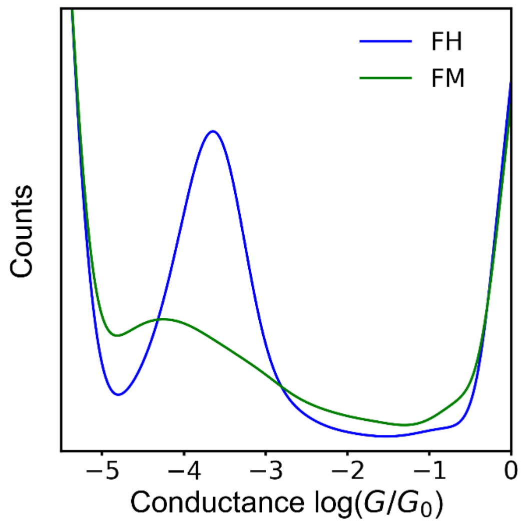

Supplementary Figure 14: 1D conductance histogram for **FH** and **FM** in propylene carbonate (PC). Data were obtained using 0.1 mM concentrations of **FH** and **FM** in propylene carbonate (PC) solvent at 250 mV applied bias across ensembles of at least 5000 single molecules.

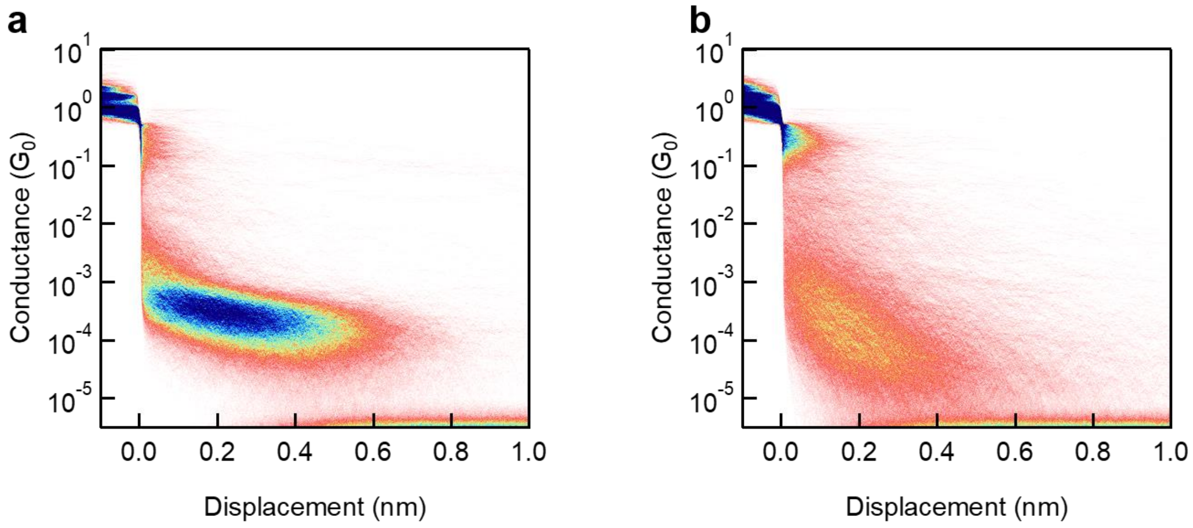

Supplementary Figure 15: 2D conductance histogram for **FH** and **FM** in propylene carbonate (PC). Data were obtained using 0.1 mM concentrations of **FH** and **FM** in propylene carbonate (PC) solvent at 250 mV applied bias across ensembles of at least 5000 single molecules.

Supplementary Table 1: Junction formation probabilities for **FH** and **FM** in 1,2,4-trichlorobenzene (TCB) and propylene carbonate (PC) solvent. The junction formation probability is defined as the ratio of the number of successful molecular junctions to the total number of traces.

Molecule	Junction formation probability (%)
**FH** (TCB)	72%
**FM** (TCB)	53%
**FH** (PC)	75%
**FM** (PC)	34%

S5. Density functional theory (DFT) calculations

S5.1. FH

N-H trans B97D/6-311G(d,p)

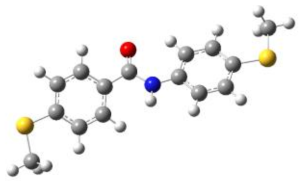

SCF Done: E(RB97D) = -1506.71253621 A.U.
Temperature 298.150 Kelvin. Pressure 1.00000 Atm.
Sum of electronic and thermal Energies= -1506.434748
Sum of electronic and thermal Enthalpies= -1506.433804
Sum of electronic and thermal Free Energies= -1506.504106
Number of imaginary frequencies: 0

Standard orientation:

Center Number	Atomic Number	Atomic Type	Coordinates (Angstroms) X	Y	Z
1	6	0	-1.896832	0.605914	-0.005153
2	6	0	-2.430924	-0.646293	-0.356883
3	6	0	-3.809684	-0.880695	-0.329710
4	6	0	-4.692611	0.145047	0.055923
5	6	0	-4.163212	1.411175	0.393463
6	6	0	-2.791361	1.635190	0.351868
7	6	0	-0.429095	0.939393	-0.042199
8	8	0	-0.032279	2.091665	-0.193169
9	7	0	0.413162	-0.152303	0.120803
10	6	0	1.823078	-0.206169	0.099780
11	6	0	2.428116	-1.449404	0.383826
12	6	0	3.811770	-1.594562	0.372382
13	6	0	4.642784	-0.495124	0.073477
14	6	0	4.039271	0.741905	-0.209700
15	6	0	2.647629	0.893342	-0.198581
16	1	0	-1.778706	-1.449358	-0.700339
17	1	0	-4.184570	-1.857445	-0.622567
18	1	0	-4.833098	2.219749	0.683874
19	1	0	-2.382482	2.614015	0.592289
20	1	0	-0.034082	-1.020676	0.384192
21	1	0	1.801838	-2.312358	0.615625
22	1	0	4.248422	-2.567055	0.596403
23	1	0	4.645597	1.612973	-0.443106
24	1	0	2.199289	1.856178	-0.410778

25	16	0	6.405810	-0.782305	0.084570
26	16	0	-6.460669	-0.035315	0.138343
27	6	0	7.088459	0.857671	-0.349077
28	1	0	6.814728	1.609109	0.401880
29	1	0	8.177257	0.732341	-0.357743
30	1	0	6.750533	1.173984	-1.343707
31	6	0	-6.727436	-1.769503	-0.378150
32	1	0	-6.235558	-2.465816	0.312103
33	1	0	-7.811565	-1.924812	-0.339240
34	1	0	-6.373471	-1.933425	-1.403354

extracted eigenvalues including HOMO and LUMO; B97D/STO-3G//B97D/6-311G(d,p)

Alpha occ. eigenvalues:	-0.19956	-0.18801	-0.15905	-0.13629	-0.12650
Alpha occ. eigenvalues:	-0.10274				
Alpha virt. eigenvalues:	0.06087	0.08822	0.09951	0.10973	0.16698

S5.2. FM

N-Me cis B97D/6-311G(d,p)

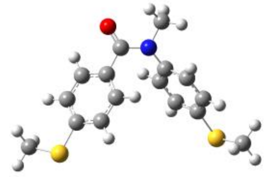

SCF Done: E(RB97D) = -1545.99653072 A.U.
Temperature 298.150 Kelvin. Pressure 1.00000 Atm.
Sum of electronic and thermal Energies= -1545.690748
Sum of electronic and thermal Enthalpies= -1545.689804
Sum of electronic and thermal Free Energies= -1545.763621
Number of imaginary frequencies: 0

Standard orientation:

Center Number	Atomic Number	Atomic Type	Coordinates (Angstroms) X	Y	Z
1	6	0	1.325200	0.596935	-1.144137
2	6	0	2.071874	-0.566080	-1.320576
3	6	0	3.050957	-0.940904	-0.376854
4	6	0	3.258142	-0.119755	0.747263
5	6	0	2.491296	1.036020	0.927293
6	6	0	1.513977	1.404454	-0.008434
7	7	0	0.735648	2.585577	0.193665
8	6	0	1.464533	3.866544	0.214153

9	6	0	-0.640359	2.648876	-0.037409
10	8	0	-1.192009	3.718858	-0.291610
11	6	0	-1.438258	1.380551	0.091642
12	6	0	-1.126639	0.344618	0.994858
13	6	0	-1.952709	-0.769479	1.117058
14	6	0	-3.115127	-0.888344	0.324611
15	6	0	-3.439154	0.149190	-0.570447
16	6	0	-2.614198	1.272334	-0.668240
17	1	0	0.575047	0.878872	-1.879285
18	1	0	1.901523	-1.182121	-2.202668
19	1	0	4.003425	-0.377066	1.494945
20	1	0	2.640637	1.650133	1.814024
21	1	0	2.403395	3.730487	0.761309
22	1	0	0.845776	4.623911	0.704125
23	1	0	1.688590	4.205725	-0.809360
24	1	0	-0.234586	0.411753	1.611954
25	1	0	-1.697008	-1.552939	1.829527
26	1	0	-4.332654	0.094235	-1.186309
27	1	0	-2.878423	2.089913	-1.335824
28	16	0	3.953022	-2.445395	-0.701810
29	16	0	-4.086312	-2.365455	0.539407
30	6	0	-5.487121	-2.113026	-0.609465
31	1	0	-5.137346	-2.037070	-1.646333
32	1	0	-6.119351	-3.001964	-0.503486
33	1	0	-6.063703	-1.221057	-0.335524
34	6	0	5.114429	-2.541664	0.707311
35	1	0	4.574847	-2.625611	1.658838
36	1	0	5.702847	-3.451593	0.543128
37	1	0	5.785086	-1.673534	0.724846

extracted eigenvalues including HOMO and LUMO; B97D/STO-3G//B97D/6-311G(d,p)

Alpha occ. eigenvalues:	-0.18851	-0.15031	-0.12746	-0.11782	-0.11286
Alpha virt. eigenvalues:	0.07215	0.09076	0.09491	0.10048	0.16379

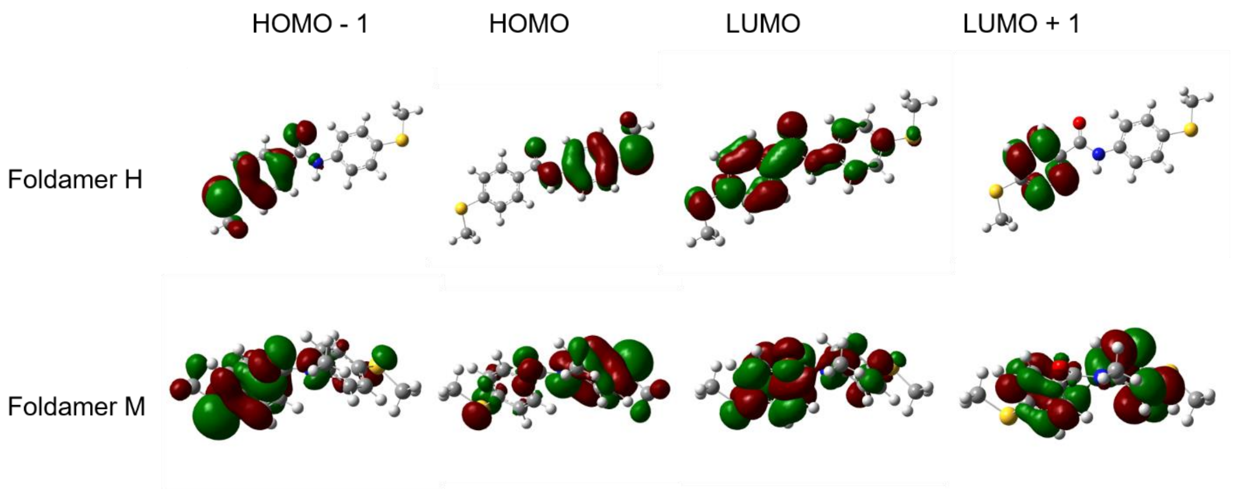

Supplementary Figure 16: Molecular orbital analysis for foldamer **FH** and **FM**. HOMO-1, HOMO, LUMO, and LUMO+1 are depicted in the figure above with an isosurface value of 0.02.

Supplementary Table 2: Molecular orbital energy values (eV) for foldamer **FH** and foldamer **FM**.

Sequence	HOMO-1	HOMO	LUMO	LUMO+1
Foldamer **FH**	-5.33	-4.70	-1.99	-1.37
Foldamer **FM**	-5.13	-4.90	-1.76	-1.34

S6. References

1. Venkataraman, Latha, Jennifer E. Klare, Iris W. Tam, Colin Nuckolls, Mark S. Hybertsen, and Michael L. Steigerwald. "Single-molecule circuits with well-defined molecular conductance." *Nano Letters* **6**, 458-462 (2006).

2. Li, Songsong, Hao Yu, Kenneth Schwieter, Kejia Chen, Bo Li, Yun Liu, Jeffrey S. Moore, and Charles M. Schroeder. "Charge transport and quantum interference effects in oxazole-terminated conjugated oligomers." *Journal of the American Chemical Society* **141**, 16079-16084 (2019).

3. Li, Bo, Hao Yu, Elena C. Montoto, Yun Liu, Songsong Li, Kenneth Schwieter, Joaquín Rodríguez-López, Jeffrey S. Moore, and Charles M. Schroeder. "Intrachain charge transport through conjugated donor–acceptor oligomers." *ACS Applied Electronic Materials* **1**, 7-12 (2018).

4. Nagahara, L. A., T. Thundat, and S. M. Lindsay. "Preparation and characterization of STM tips for electrochemical studies." *Review of scientific instruments* **60**, 3128-3130 (1989).

5. Adak, Olgun, Ethan Rosenthal, Jeffery Meisner, Erick F. Andrade, Abhay N. Pasupathy, Colin Nuckolls, Mark S. Hybertsen, and Latha Venkataraman. "Flicker noise as a probe of electronic interaction at metal–single molecule interfaces." *Nano letters* **15**, 6, 4143-4149 (2015).

6.Frisch, M. E., Trucks, G. W., Schlegel, H. B., Scuseria, G. E., Robb, M., Cheeseman, J. R., ... & Fox, D. J. Gaussian 16 (2016).

7. Brandbyge, Mads, José-Luis Mozos, Pablo Ordejón, Jeremy Taylor, and Kurt Stokbro. "Density-functional method for nonequilibrium electron transport." *Physical Review B* **65**, 165401 (2002).

8. Soler, José M., Emilio Artacho, Julian D. Gale, Alberto García, Javier Junquera, Pablo Ordejón, and Daniel Sánchez-Portal. "The SIESTA method for ab initio order-Nmaterials simulation." *Journal of Physics: Condensed Matter* **14**, 2745 (2002).

9. Papior, Nick, Nicolás Lorente, Thomas Frederiksen, Alberto García, and Mads Brandbyge. "Improvements on non-equilibrium and transport Green function techniques: The next-generation transiesta." *Computer Physics Communications* **212**, 8-24 (2017).

10. Perdew, John P., Kieron Burke, and Matthias Ernzerhof. "Generalized gradient approximation made simple." *Physical review letters* **77**, 3865 (1996).

11. Batra, Arunabh, Pierre Darancet, Qishui Chen, Jeffrey S. Meisner, Jonathan R. Widawsky, Jeffrey B. Neaton, Colin Nuckolls, and Latha Venkataraman. "Tuning rectification in single-molecular diodes." *Nano Letters* **13**, 6233-6237 (2013).
